# Longitudinal Association of Total Tau Concentrations and Physical Activity With Cognitive Decline in a Population Sample

**DOI:** 10.1001/jamanetworkopen.2021.20398

**Published:** 2021-08-11

**Authors:** Pankaja Desai, Denis Evans, Klodian Dhana, Neelum T. Aggarwal, Robert S. Wilson, Elizabeth McAninch, Kumar B. Rajan

**Affiliations:** 1 Rush Institute for Healthy Aging, Rush University Medical Center, Chicago, Illinois; 2 Rush Alzheimer’s Disease Center, Rush University Medical Center, Chicago, Illinois; 3 Department of Neurological Sciences, Rush University Medical Center, Chicago, Illinois; 4 Department of Neurology, University of California at Davis, Davis

## Abstract

**Importance:**

Tau is a brain protein located in neurons and develops abnormally in individuals with Alzheimer disease. New technology is convenient for measuring blood total tau concentrations and provides a unique and increased opportunity for early intervention to slow cognitive decline.

**Objective:**

To evaluate the association of physical activity and total tau concentrations with cognitive decline at baseline and over time.

**Design, Setting, and Participants:**

The Chicago Health and Aging Project is a population-based cohort study conducted in 4 Chicago communities. Data collection occurred in 3-year cycles between 1993 and 2012. Participants completed in-home interviews. Clinical evaluations, which included blood samples, were performed with a stratified random sample of 1159 participants. Statistical analyses were conducted from October 30, 2020, to May 25, 2021.

**Exposures:**

Physical activity and total serum tau concentrations. Data on physical activity were obtained through self-report items, and a sum of minutes per week was calculated. Little physical activity was defined as no participation in a minimum of 4 of the items on the physical activity measure. Medium activity was defined as participating in less than 150 minutes of physical activity per week, and high activity was defined as participating in 150 minutes or more of physical activity per week.

**Main Outcomes and Measures:**

The main outcome for this study is global cognitive function, measured through a battery of cognitive tests. The study hypothesis was developed after data were collected.

**Results:**

Of the 1159 participants in the study, 728 were women (63%), and 696 were African American (60%); the mean (SD) age was 77.4 (6.0) years, and the mean (SD) educational level was 12.6 (3.5) years. Participants with high total tau concentrations with medium physical activity had a 58% slower rate of cognitive decline (estimate, –0.028 standard deviation unit [SDU] per year [95% CI, –0.057 to 0.002 SDU per year]; difference, 0.038 SDU per year [95% CI, 0.011-0.065 SDU per year]), and those with high physical activity had a 41% slower rate of cognitive decline (estimate, –0.038 SDU per year [95% CI, –0.068 to –0.009 SDU per year]; difference, 0.027 SDU per year [95% CI, –0.002 to 0.056 SDU per year]), compared with those with little physical activity. Among participants with low total tau concentrations, medium physical activity was associated with a 2% slower rate of cognitive decline (estimate, –0.050 SDU per year [95% CI, –0.069 to –0.031 SDU per year]; difference, 0.001 SDU per year [95% CI, –0.019 to 0.021 SDU per year]), and high physical activity was associated with a 27% slower rate of cognitive decline (estimate, –0.037 SDU per year [95% CI, –0.055 to –0.019 SDU per year]; difference, 0.014 SDU per year [95% CI, –0.007 to 0.034 SDU per year]), compared with little physical activity. Individual tests of cognitive function showed similar results.

**Conclusions and Relevance:**

This study suggests that, among participants with both high and low total tau concentrations, physical activity was associated with slower cognitive decline. Results support the potential utility of blood biomarkers in measuring the benefits associated with health behaviors and may contribute to specifying target populations or informing interventions for trials that focus on improving physical activity behavior. Future work should examine the association of total tau concentrations with other health behaviors and physical activity types.

## Key Points

**Question:**

Is physical activity associated with slower cognitive decline in people with higher total tau concentrations?

**Findings:**

In this population-based cohort study comprising 1159 participants, medium physical activity was associated with a 58% slower rate of cognitive decline, and high physical activity was associated with a 41% slower rate of cognitive decline compared with little physical activity among those with high total tau concentrations. For participants with low total tau concentrations, medium physical activity was associated with a 2% slower rate of cognitive decline, and high physical activity was associated with a 27% slower rate of cognitive decline compared with little physical activity.

**Meaning:**

This study suggests that measurement of blood biomarkers may offer an opportunity for early intervention of physical activity to reduce the rate of cognitive decline.

## Teaser

This cohort study uses data from the Chicago Health and Aging Project to evaluate the association of physical activity and total tau concentrations with cognitive decline at baseline and over time

## Introduction

Tau is abnormally phosphorylated in the brains of individuals with Alzheimer disease (AD) and serves as a potential target for treatment or intervention.^1^ However, more research is needed to understand the role of tau in the progression of neurodegenerative disease.^2^ Examination of tau levels has generally been conducted using cerebrospinal fluid (CSF). In recent years, tau measurement in blood using a single molecular array has provided a stable assessment of tau concentrations in the human brain.^3,4^ Blood assays offer a less intrusive and more cost-effective way of measuring total tau concentrations. Increased concentrations of total tau in plasma are associated with cognitive decline,^5^ specifically in memory, cognition, visuospatial capacity, attention, and progression from mild cognitive impairment to AD,^6,7^ and distinguish between individuals with AD and controls.^8^ Higher levels of plasma total tau are correlated with amnesic mild cognitive impairment, specifically, and associated with decline in cognitive function and atrophy of the brain,^9^ especially low hippocampal volume and increased neurofibrillary tangles.^10^ However, examining whether preventive factors in individuals with a high total tau concentration are associated with slower cognitive decline is an important question to address for disease prevention.

A lengthy preclinical AD phase offers the chance for intervention,^11^ such as engagement in physical activity or increasing adherence to physical activity, to positively impact the disease before clinical symptom onset.^12,13^ However, limited work has been done to understand the association of physical activity and total tau concentrations with cognitive decline. In general, physical activity may guard against cognitive decline, decrease the chance of developing AD, and slow dementia onset.^14,15,16^ Increased physical activity is also associated with higher hippocampal and total brain volumes^17^ and weakened neuroimaging biomarkers associated with age.^18^ Increased physical activity mitigates cognitive decline owing to β-amyloid burden and gray matter volume deficit, measured through magnetic resonance imaging.^13^ However, it is not known whether physical activity mitigates cognitive decline in people with higher tau concentrations. We addressed this question in a biracial population-based sample with total tau concentrations, physical activity assessments, and longitudinal changes in cognitive function.

## Methods

The Chicago Health and Aging Project (CHAP) is a population-based cohort study of African American and White participants older than 65 years in 4 Chicago communities, recruited through door-to-door census. Data collection for the study as a whole occurred in 3-year cycles between 1993 and 2012, consisting of 4 cohorts, which met the age requirement over the study duration. The CHAP study has current approval by the institutional review board at the Rush University Medical Center. Written informed consent was obtained from all study participants. This study followed the Strengthening the Reporting of Observational Studies in Epidemiology (STROBE) reporting guideline for observational studies.

Data collection included in-home interviews, and a stratified random sample, approximately one-third of participants, took part in clinical evaluations in which blood samples were collected. In total, there were 11 600 blood samples collected among 5696 participants. Owing to budget constraints, immunoassays were conducted on 3000 samples.^5,11^ For this analysis, 1159 participants with a baseline blood sample collected and at least 2 global cognitive function outcome measurements were selected. Race/ethnicity was assessed by participants’ responses to the 1990 US Census questions^19^; it was measured because of its importance to the risks, consequences, and distributions of common chronic diseases among older people. Study participants were aged 65 years or older and did not have AD at baseline.^20^

### Measurement of Total Tau Concentrations

Blood samples were put on dry ice and transported by study team members to the Rush Biorepository freezer and stored at −80 °C between 1994 and 2012. In 2019, previously unthawed blood samples were sent to Quanterix Corporation and the total tau concentration was assayed in duplicates through the single molecular assay bead-based HD platform and the Neurology 4Plex A kit.^5^ The mean concentration of total tau of duplicate measurements was used in analysis, and the coefficient of variation among duplicates was 7.3%. For this analysis, we used the first (baseline) assessment of total tau concentration available for 1159 participants.

### Physical Activity

Information on physical activity was obtained through 1985 US Health Interview Survey items. Participants reported the frequency (instances) and duration (in minutes) of participation in walking for exercise, jogging or running, gardening or yard work, dancing, calisthenics or general exercise, golf, bowling, bicycle riding, swimming or water exercises, other exercises, sports, or physically active hobbies in the past 14 days.^21,22^ Total physical activity participation was then converted into minutes per week (7 days per week) by multiplying the number of instances by duration and dividing by 2. Total physical activity participation was calculated as hours per week by dividing minutes per week by 60 minutes.

### Cognitive Function Battery

Cognitive tests were conducted during in-home assessments. Global cognitive function was measured through the East Boston Tests of Immediate Memory and Delayed Recall (episodic memory), the Mini-Mental State Examination (MMSE), and the Symbol Digit Modalities Test (modified, oral version) (perceptual speed).^23,24,25,26^ The *z* scores were calculated for each test, using baseline means and SDs for the complete CHAP sample. Global cognitive function was calculated by taking the mean of the *z* scores for the tests.^27^

### Statistical Analysis

Statistical analysis was conducted from October 30, 2020, to May 25, 2021, using SAS, version 9.4 (SAS Institute Inc). Baseline descriptive analysis was performed to understand the sample characteristics of CHAP participants who completed 2 or more global cognitive assessment measures and had at least 1 blood sample collected. Mixed-effects regression analyses were conducted, beginning with the time of first blood sample collection and using all the interviews that followed, to test the association of independent variables—physical activity and total tau concentrations—with the following dependent variables: global composite cognitive function and individual tests of episodic memory, perceptual speed, and the MMSE. Models were conducted with physical activity and total tau concentrations as continuous as well as categorical variables. Continuous models were conducted with physical activity in hours per week, and categorical models were conducted with physical activity in minutes per week. For categorical models, physical activity was organized into 3 groups: little activity, medium activity, and high activity. Participants in the little activity group had to respond to at least 4 of the activity-specific items on the physical activity measure, and all responses had to indicate that they had not participated (0 minutes per week) in the specified activities. Medium activity was defined as participating in activities for less than 150 minutes per week, and high activity was defined as 150 minutes or more of activities per week. This cutoff of 150 minutes per week was determined based on the 2018 *Physical Activity Guidelines for Americans*, 2nd Edition.^28^ Total tau concentration was categorized into 2 groups: low (≤0.40 pg/mL) and high (>0.40 pg/mL). Previous research showed that individuals with a total tau concentration above 0.40 pg/mL showed a markedly faster rate of cognitive decline than those with a total tau concentration of 0.40 pg/mL or less.^5^ All models were adjusted for age, race/ethnicity, sex, educational level, chronic medical conditions, and apolipoprotein e4 and each of their interactions with time. We created a variable for weather based on the month that data collection occurred. December to April was coded as 1, and March to November was coded as 0. The regression models also include person-specific intercept and slopes and an unstructured correlation matrix structure. Generally, comparisons made in this study were planned. Bonferonni corrections were conducted to correct for multiple comparisons. Mixed effects regression models were conducted separately for female and male participants and for White and African American participants, with global cognitive function as the outcome. Plots were generated using R, version 4.0.3 (R Group for Statistical Computing, Vienna). A priori levels of significance include *P* < .05 and *P* < .01. Hypothesis tests were 2-sided.

## Results

Table 1 describes the baseline characteristics of 1159 CHAP study participants included in this study, who completed 3507 cognitive assessments and had at least 1 total tau concentration measurement. The mean (SD) number of observations per participant was 3, with a minimum of 2 and a maximum of 6 observations. Measurement duration for this analysis was from 1994 to 2012. The study sample consisted of 728 women (63%) and 696 African American individuals (60%); the mean (SD) age was 77.4 (6.0) years and mean (SD) educational level was 12.6 (3.5) years. Among the 1159 participants, 357 (31%) reported little physical activity; 400 (35%) reported less than 150 minutes per week of physical activity (medium), and 402 (35%) reported 150 minutes per week or more of physical activity (high). Participants with medium activity spent a median of 62.5 minutes per week (range, 2-150 minutes) engaged in physical activity, and those with high activity spent a median of 327.5 minutes per week (range, 153-2940 minutes) engaged in physical activity. The geometric mean of the concentration of total tau was 0.48 pg/mL, with a lower limit of detection of 0.096 pg/mL. The Spearman correlation between total tau and age at baseline was *r* = 0.132. The eFigure in the Supplement describes the number of participants at baseline within the high vs low total tau concentration grouping, by physical activity category. The low total tau concentration group had more active participants than the high total tau concentration group. The number of inactive participants was similar for the high and low total tau concentration groups. eTable 1 in the Supplement shows the results of the mixed-effects regression models with physical activity and total tau concentration as continuous variables. The interaction of physical activity, total tau concentration, and time was not statistically significant for all cognitive outcomes. eTables 2 and 3 in the Supplement show findings of categorical mixed-effects regression models examining physical activity stratified by total tau concentration and cognitive outcomes. The interaction of medium physical activity with time (β = 0.038; *P* = .006) was statistically significant for global cognitive function among participants with high total tau concentrations. The interaction of medium physical activity (β = 0.031; *P* = .03) and high physical activity (β = 0.031; *P* = .03) with time were both statistically significant for perceptual speed among those with high total tau concentrations. The interaction of high physical activity with time (β = 0.025; *P* = .03) was statistically significant for MMSE score among those with low total tau concentrations. For participants with high total tau concentrations, the interaction of medium physical activity with time (β = 0.047; *P* = .007) was statistically significant for MMSE scores. Generally, comparisons made in this study were planned. Bonferroni corrections were conducted to correct for multiple comparisons. Mixed-effects regression models were conducted separately for female and male participants and for White and African American participants with global cognitive function as the outcome. We did not find statistically significant differences in the moderation of physical activity in the association of total tau concentration with global cognitive function in all of these models. We created a variable for weather based on the month that data collection occurred. December to April was coded as 1 and March to November was coded as 0. Although physical activity level was different in winter months, weather was not statistically significant in the models.

**Table 1.** Baseline Characteristics of Participants

Characteristic	Participants (N = 1159)
Age, mean (SD), y	77.4 (6.0)
Educational level, mean (SD), y	12.6 (3.5)
Sex, No. (%)	
Female	728 (63)
Male	431 (37)
African American, No. (%)	696 (60)
Chronic medical conditions, mean (SD), No.	1.3 (1.0)
*APOE4*, No. (%)	393 (34)
Composite physical activity score, mean (SD), min/wk	170.6 (4.5)
Composite physical activity score by activity level, min/wk	
Little activity (response to at least 4 items on physical activity measure is 0 min/wk)	
No. participants	357
Median (range)	0
Mean (SD)	0
Medium activity (<150 min/wk)	
No. participants	400
Median (range)	62.5 (2 to 150)
Mean (SD)	71 (42)
High activity (≥150 min/wk)	
No. participants	402
Median (range)	327.5 (153 to 2940)
Mean (SD)	422 (330)
Total tau, geometric, mean (95% CI), pg/mL	
Low total tau (≤0.40 pg/mL)	0.48 (0.47 to 0.50)
High total tau (>0.40 pg/mL)	0.48 (0.47 to 0.50)
Global cognitive function score	
Mean (SD)	0.209 (0.680)
Range	(–3.026 to 1.456)
Episodic memory score	
Mean (SD)	0.189 (0.827)
Range	(–2.462 to 1.418)
Mini-Mental State Examination score	
Mean (SD)	0.257 (0.609)
Range	(–4.143 to 0.810)
Perceptual speed score	
Mean (SD)	0.245 (0.902)
Range	(–1.747 to 2.800)

Abbreviation: *APOE4*, apolipoprotein e4.

### Baseline Cognitive Function

Table 2 shows the association of physical activity and global cognitive function at baseline, stratified by total tau concentration. Among participants with low concentrations of total tau (≤0.40 pg/mL), we found that the baseline global cognitive function score was 47% higher for those with medium physical activity (standard deviation units [SDUs] or β = 0.411 [95% CI, 0.289-0.533 SDU]; difference, 0.131 SDU [95% CI, 0.015-0.248 SDU]) and 41% higher for those with high physical activity (0.394 SDU [95% CI, 0.282-0.506 SDU]; difference, 0.115 SDU [95% CI, –0.006 to 0.235 SDU]) compared with those with little physical activity (0.280 SDU [95% CI, 0.146-0.414]). For participants with high total tau concentrations (>0.40 pg/mL), participants with medium physical activity (0.478 SDU [95% CI, 0.330-0.626 SDU]; difference, 0.037 [95% CI, –0.090 to 0.163 SDU]) and participants with high physical activity (0.479 SDU [95% CI, 0.332-0.625 SDU]; difference, 0.037 [95% CI, –0.010 to 0.174 SDU]) both had 8% better cognitive function at baseline compared with participants with little physical activity (0.442 SDU [95% CI, 0.289-0.594 SDU]). eTable 4 in the Supplement generally shows similar findings for individual tests of cognitive function, where participants with medium physical activity and high physical activity had better scores at baseline compared with participants with little physical activity within both total tau concentration groups.

**Table 2.** Association of Physical Activity With Baseline Level of Global Cognitive Function Stratified by Concentration of Total Tau^a^

Characteristic	Standard deviation units (95% CI)		Difference, %
	Estimate	Difference	
Low tau concentration			
Little physical activity	0.280 (0.146 to 0.414)	1 [Reference]	1 [Reference]
Medium physical activity	0.411 (0.289 to 0.533)	0.131 (0.015 to 0.248)	47
High physical activity	0.394 (0.282 to 0.506)	0.115 (–0.006 to 0.235)	41
High tau concentration			
Little physical activity	0.442 (0.289 to 0.594)	1 [Reference]	1 [Reference]
Medium physical activity	0.478 (0.330 to 0.626)	0.037 (–0.090 to 0.163)	8
High physical activity	0.479 (0.332 to 0.625)	0.037 (–0.010 to 0.174)	8

^a^ Physical activity: little activity (responded to at least 4 items and reported little activity for all responses), medium activity (<150 min/wk), and high activity (>150 min/wk). Total tau: low (≤0.40 pg/mL) and high (>0.40 pg/mL). All models adjusted for age, race/ethnicity, sex, educational level, chronic medical conditions, and apolipoprotein e4.

### Rate of Cognitive Decline

Table 3 shows the annual rate of decline in global cognitive function for participants with low vs high total tau concentrations. Among participants with high total tau concentrations, the rate of cognitive decline for participants with medium physical activity was 58% slower (−0.028 SDU per year [95% CI, −0.057 to 0.002 SDU per year]; difference, 0.038 SDU per year [95% CI, 0.011-0.065 SDU per year]), and the rate of cognitive decline for participants with high physical activity was 41% slower (−0.038 SDU per year [95% CI, −0.068 to −0.009 SDU per year]; difference, 0.027 [95% CI, −0.002-0.056 SDU per year]), compared with those with little physical activity (−0.066 SDU per year [95% CI, −0.097 to −0.034 SDU per year]). Thus, participants with high concentrations of total tau with low and high physical activity showed significantly slower cognitive decline compared with those with little physical activity.

**Table 3.** Association of Physical Activity With Rate of Decline in Global Cognition Stratified by Concentration of Total Tau^a^

Characteristic	Standard deviation units (95% CI)		Difference, %
	Estimate	Difference	
Low tau concentration			
Little physical activity	–0.051 (–0.072 to –0.029)	1 [Reference]	1 [Reference]
Medium physical activity	–0.050 (–0.069 to –0.031)	0.001 (–0.019 to 0.021)	2
High physical activity	–0.037 (–0.055 to –0.019)	0.014 (–0.007 to 0.034)	27
High tau concentration			
Little physical activity	–0.066 (–0.097 to –0.034)	1 [Reference]	1 [Reference]
Medium physical activity	–0.028 (–0.057 to 0.002)	0.038 (0.011 to 0.065)	58
High physical activity	–0.038 (–0.068 to –0.009)	0.027 (–0.002 to 0.056)	41

^a^ Physical activity: little activity (responded to at least 4 items and reported little activity for all responses), medium activity (<150 min/wk), and high activity (>150 min/wk). Total tau: low (≤0.40 pg/mL) and high (>0.40 pg/mL). All models adjusted for age, race/ethnicity, sex, educational level, chronic medical conditions, and apolipoprotein e4 and each of their interactions with time.

Among participants with low total tau concentrations, medium physical activity was associated with a 2% slower rate of cognitive decline (–0.050 SDU per year [95% CI, –0.069 to –0.031 SDU per year]; difference, 0.001 SDU per year [95% CI, –0.019 to 0.021 SDU per year]), and high physical activity was associated with a 27% slower rate of cognitive decline (–0.037 SDU per year [95% CI, –0.055 to –0.019 SDU per year]; difference, 0.014 SDU per year [95% CI, –0.007 to 0.034 SDU per year]), compared with little physical activity (–0.051 SDU per year [95% CI, –0.072 to –0.029 SDU per year] (Table 3). Thus, participants with low concentrations of total tau with high physical activity showed significantly slower cognitive decline compared with those with no or medium physical activity.

The Figure depicts longitudinal changes in cognitive function measures by total tau concentrations and physical activity level. The plots show steep declines in cognitive function among participants with little physical activity and high total tau concentrations. This group had the lowest scores over time compared with the other physical activity and total tau concentration groups. Participants with high physical activity and low total tau concentrations tended to maintain high cognitive function scores over time.

**Figure.**
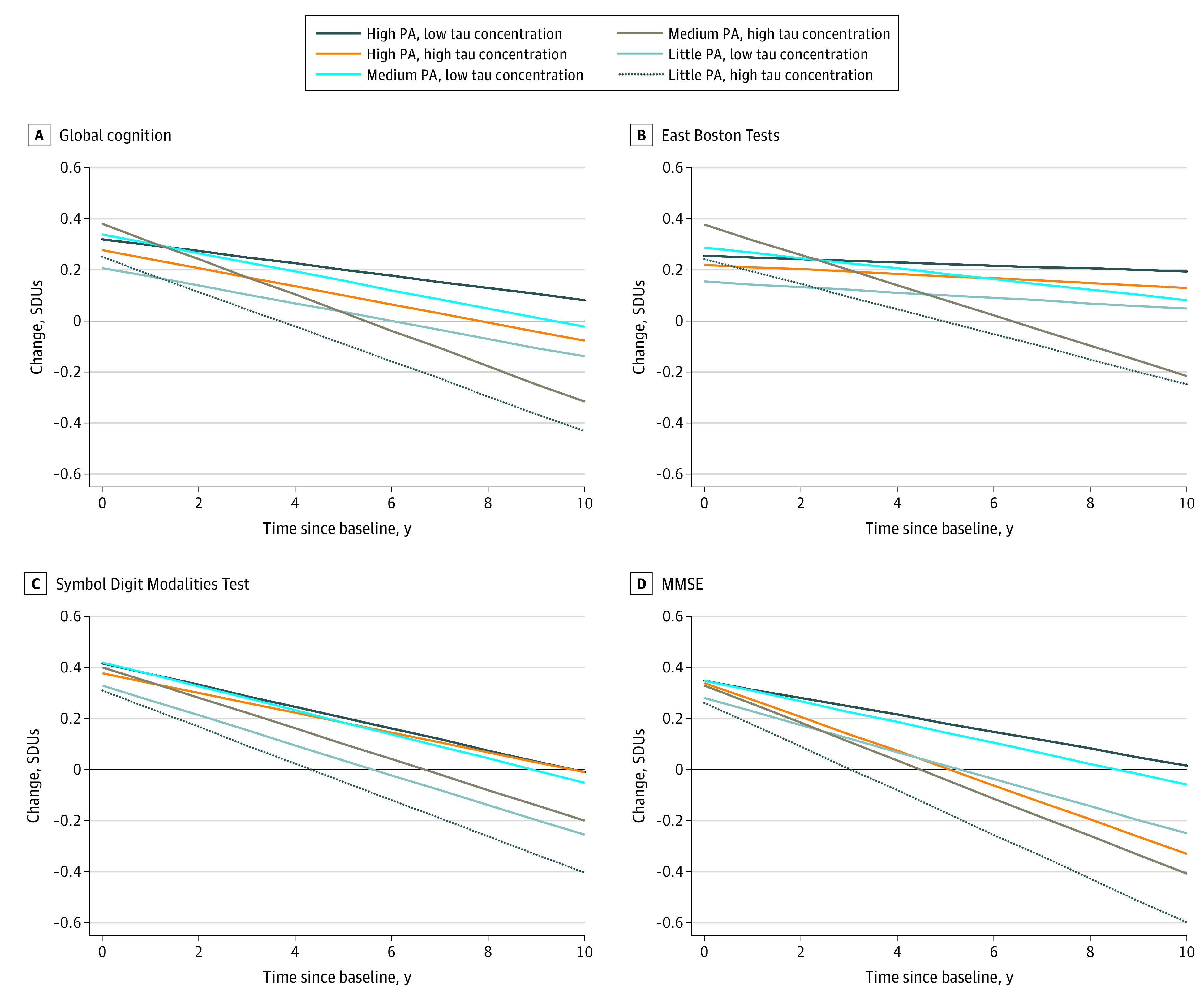
Annual Change in Cognition by Physical Activity and Total Tau Concentration Group A, Global cognition summary measure. B, East Boston Tests of Immediate Memory and Delayed Recall. C, Symbol Digit Modalities Test. D, Mini-Mental State Examination (MMSE). PA indicates physical activity; SDUs, standard deviation units.

Table 4 describes the annual rate of decline in the episodic memory, perceptual speed, and MMSE score. Similar to global cognitive function results, participants with medium and high physical activity had slower rates of decline in episodic memory, perceptual speed, and MMSE score compared with those with little physical activity for both total tau concentration groups. For episodic memory among participants with high total tau concentrations, those with medium physical activity had a 74% decline (–0.009 SDU [95% CI, –0.043 to 0.026 SDU]; difference, 0.026 SDU [95% CI, –0.007 to 0.060 SDU]), and those with high physical activity had more than 100% decline (0.0005 SDU [95% CI, –0.035 to 0.036 SDU]; difference, 0.035 SDU [95% CI, 0-0.071 SDU]), compared with those with little physical activity. Among participants with low total tau concentrations, those with medium physical activity had a 38% decline in episodic memory (–0.029 SDU [95% CI, –0.053 to –0.005 SDU]; difference, –0.008 SDU [95% CI, –0.033 to 0.018 SDU]), and those with high physical activity had a 33% decline in episodic memory (–0.015 SDU [95% CI, –0.037 to 0.007 SDU]; difference, 0.007 SDU [95% CI, –0.019 to 0.032 SDU]), compared with participants with little physical activity.

**Table 4.** Rate of Decline for Episodic Memory, Perceptual Speed, and Mini-Mental State Examination Score^a^

Characteristic	Standard deviation units (95% CI)		Difference, %
	Estimate	Difference	
**Episodic memory**			
Low tau concentration			
Little physical activity	–0.021 (–0.049 to 0.006)	1 [Reference]	1 [Reference]
Medium physical activity	–0.029 (–0.053 to –0.005)	–0.008 (–0.033 to 0.018)	38
High physical activity	–0.015 (–0.037 to 0.007)	0.007 (–0.019 to 0.032)	33
High tau concentration			
Little physical activity	–0.035 (–0.073 to 0.003)	1 [Reference]	1 [Reference]
Medium physical activity	–0.009 (–0.043 to 0.026)	0.026 (–0.007 to 0.060)	74
High physical activity	0.0005 (–0.035 to 0.036)	0.035 (0 to 0.071)	>100
**Perceptual speed**			
Low tau concentration			
Little physical activity	–0.070 (–0.092 to –0.047)	1 [Reference]	1 [Reference]
Medium physical activity	–0.059 (–0.079 to –0.039)	0.010 (–0.011 to 0.032)	14
High physical activity	–0.054 (–0.072 to –0.035)	0.016 (–0.005 to 0.037)	23
High tau concentration			
Little physical activity	–0.091 (–0.122 to –0.061)	1 [Reference]	1 [Reference]
Medium physical activity	–0.061 (–0.089 to –0.033)	0.031 (0.004 to 0.057)	34
High physical activity	–0.061 (–0.089 to –0.032)	0.031 (0.002 to 0.059)	34
**Mini-Mental State Examination score**			
Low tau concentration			
Little physical activity	–0.077 (–0.102 to –0.051)	1 [Reference]	1 [Reference]
Medium physical activity	–0.062 (–0.084 to –0.039)	0.015 (–0.008 to 0.038)	19
High physical activity	–0.051 (–0.072 to –0.030)	0.025 (0.002 to 0.049)	32
High tau concentration			
Little physical activity	–0.077 (–0.118 to –0.037)	1 [Reference]	1 [Reference]
Medium physical activity	–0.030 (–0.069 to 0.009)	0.047 (0.013 to 0.082)	61
High physical activity	–0.063 (–0.102 to –0.025)	0.014 (–0.023 to 0.051)	18

^a^ Physical activity: little activity (responded to at least 4 items and reported little activity for all responses), medium activity (<150 min/wk), and high activity (>150 min/wk). Total tau: low (≤0.40 pg/mL) and high (>0.40 pg/mL). All models adjusted for age, race/ethnicity, sex, educational level, chronic medical conditions, and apolipoprotein e4 and each of their interactions with time.

Regarding perceptual speed among participants with high total tau concentrations, both those with medium physical activity and those with high physical activity had a 34% decline (medium, –0.061 SDU [95% CI, –0.089 to –0.033 SDU]; difference, 0.031 SDU [95% CI, 0.004-0.057 SDU]; high, –0.061 SDU [95% CI, –0.089 to –0.032 SDU]; difference, 0.031 SDU [95% CI, 0.002-0.059 SDU]) compared with participants with little physical activity (Table 4). For perceptual speed among participants with low total tau concentrations, those with medium physical activity had a 14% decline (–0.059 SDU [95% CI, –0.079 to –0.039 SDU]; difference, 0.010 SDU [95% CI, –0.011 to 0.032 SDU]), and those with high physical activity had a 23% decline (–0.054 SDU [95% CI, –0.072 to –0.035 SDU]; difference, 0.016 SDU [95% CI, –0.005 to 0.037 SDU]). With regard to MMSE scores among participants with high total tau concentrations, those with medium physical activity had a 61% decline (–0.030 SDU [95% CI, –0.069 to 0.009 SDU]; difference, 0.047 SDU [95% CI, 0.013-0.082 SDU]), and those with high physical activity had an 18% decline (–0.063 SDU [95% CI, –0.102 to –0.025 SDU]; difference, 0.014 SDU [95% CI, –0.023 to 0.051 SDU]), compared with participants with little physical activity. Among participants with low total tau concentrations, those with medium physical activity had a 19% decline in MMSE scores (–0.062 SDU [95% CI, –0.084 to –0.039 SDU]; difference, 0.015 SDU [95% CI, –0.008 to 0.038 SDU]), and those with high physical activity had a 32% decline (–0.051 SDU [95% CI, –0.072 to –0.030 SDU]; difference, 0.025 SDU [95% CI, 0.002-0.049 SDU]) in MMSE scores compared with participants with little physical activity.

## Discussion

Our findings augment existing work examining serum biomarkers and cognitive function.^5^ At baseline, participants with little physical activity had lower cognitive function scores than those with medium physical activity and high physical activity within both total tau concentration groups. Over time, participants with medium physical activity and high physical activity had slower cognitive decline compared with participants with little physical activity within both total tau concentration groups. Although we know that physical activity can positively impact cognitive function, much less is understood about the role of sedentary behavior and its association with the same.^29^ To our knowledge, few studies have evaluated the association between physical activity and cognitive function in a sample without diagnosis of cognitive conditions. More research is necessary to assess the kind, amount, and level of activity needed to maintain cognitive function.^15^ Our results may contribute to these gaps in knowledge by demonstrating the clear difference in cognitive decline among participants with little activity, compared with those who reported low or high activity in a large, population-based sample without disease. We found that participants with little physical activity and high total tau concentrations experienced a substantial decline across all cognitive outcomes and, inversely, participants with high physical activity and low total tau concentrations tended to sustain high scores over time. These results suggest that physical activity may have a positive association with cognitive function and that sedentary behavior may have a negative association with cognitive function.

A study conducted with participants from the Wisconsin Registry for Alzheimer Prevention, who were later-middle aged, found that physical activity, measured by accelerometer, at moderate intensity had statistically significant associations with increased β-amyloid 42 (Aβ42) concentrations, decreased total tau to Aβ42 ratio concentrations, and decreased phosphorylated tau to Aβ42 ratio concentrations.^30^ A higher level of sedentary behavior was associated with decreased Aβ42 concentrations. No associations were observed for low-intensity or high-intensity physical activity and CSF biomarkers. The authors called for the need to validate their findings. Our study helps to do so by evaluating the association of physical activity and total tau concentrations with cognitive function in a large sample.

To our knowledge, ours is the first study to longitudinally evaluate physical activity in the association between total tau in serum and cognitive function. Only 1 study was found that evaluated CSF and plasma biomarkers among participants with AD.^31^ Participants were randomized to a 16-week physical activity intervention or control with pre-post evaluation. Results showed that the soluble trigger receptor expressed on myeloid cells 2 measured in CSF had a stronger association after physical activity intervention compared with before the intervention, and interleukin 6 levels measured in plasma were higher for the intervention group compared with controls after the intervention. No changes were observed in other biomarkers examined after the intervention. The authors indicate that more specific physical activity characteristics should be evaluated in future studies. Our study focuses on the frequency and duration of physical activity, contributing to filling this gap. Evaluating the associations of physical activity with AD biomarkers is an emerging area of research.^18^ Previous studies have cross-sectionally examined the association between physical activity among participants without diagnosis of neurodegenerative disease and AD biomarkers obtained from CSF with small sample sizes. An examination of CSF biomarkers Aβ42, tau, and phosphorylated tau_181_ showed that reduced physical activity had statistically significant associations with high tau and phosphorylated tau_181_ concentrations, as did increased physical activity with high Aβ42 concentrations.^32^

### Limitations and Strengths

Our study has some limitations. As with other studies on this subject, the direction of causation in the associations between physical activity and pathology of AD remains unclear, cutoffs for biomarker measurements are not well established, and use of self-report to measure physical activity has reported issues.^32^ For our study, it is difficult to know if the findings reflect the benefit associated with high physical activity or the cost of medium physical activity, which requires further exploration. Physical activity is measured through self-report, which is subject to social desirability and recall biases.^33^ Intensity of physical activity is not built into the physical activity measure.

Selection bias is also a limitation, given that a subset of the CHAP sample was used for this analysis. About one-third of the baseline sample engaged in 150 minutes or more of activity per week, which is not generalizable to the population of older adults in the US. The study sample had a high median baseline physical activity level, which consisted of a wide range of physical activity participation. At times, medium physical activity was associated with a slower rate of cognitive decline compared with high physical activity, suggesting an association that is nonmonotonic between medium and high physical activity. The reasons for this are unclear but may be related to how physical activity was categorized. The design of the study did not allow for accurate identification of specific periods of inactivity, especially if brief, or for attribution of such periods of inactivity to episodes of pain or surgery. Other racial/ethnic minorities were not represented in the study.

Our study also has some strengths. The study had a large, population-based, biracial sample, serum biomarkers were collected, and participants had at least 2 measurement points of cognitive function.

## Conclusions

In this study, physical activity was associated with slower cognitive decline among participants with both low and high concentrations of total tau. Future work should focus on examining the associations between physical activity and additional biomarkers, including phosphorylated tau_181_, phosphorylated tau_217_, and neurofilament light chain. Increased levels of these blood-based biomarkers have been shown to be associated with AD diagnosis both pathologically as well as clinically.^34^ Additional research should evaluate the associations of other health behaviors and particular types of physical activity (such as aerobic or strengthening activities) and blood biomarkers with outcomes such as cognitive function and magnetic resonance imaging results. Findings may inform the development of prevention trials or interventions that are tailored to individuals with at-risk characteristics with long-term follow-up measurement.

## Notes

Supplement. **eFigure.** Number of Participants by Physical Activity and Total Tau Group **eTable 1.** Longitudinal Association of Continuous Variables: Physical Activity and Total Tau With Cognitive Outcomes **eTable 2.** Longitudinal Association of Categorical Variables: Physical Activity and Total Tau With Global Cognitive Function **eTable 3.** Longitudinal Association of Categorical Variables: Physical Activity and Total Tau With Cognitive Outcomes **eTable 4.** Baseline Level of Episodic Memory, Perceptual Speed and MMSE
