# Supplementary material for: Longitudinal Association of Total Tau Concentrations and Physical Activity With Cognitive Decline in a Population Sample
Source: JAMA Netw Open. 2021 Aug 11;4(8):e2120398. doi: 10.1001/jamanetworkopen.2021.20398 (PMC8358733; doi:10.1001/jamanetworkopen.2021.20398)
Supplement: Supplement. — eFigure. Number of Participants by Physical Activity and Total Tau Group eTable 1. Longitudinal Association of Continuous Variables: Physical Activity and Total Tau With Cognitive Outcomes eTable 2. Longitudinal Association of Categorical Variables: Physical Activity and Total Tau With Global Cognitive Function eTable 3. Longitudinal Association of Categorical Variables: Physical Activity and Total Tau With Cognitive Outcomes eTable 4. Baseline Level of Episodic Memory, Perceptual Speed and MMSE [file jamanetwopen-e2120398-s001.pdf]

Supplementary Online Content

Desai P, Evans D, Dhana K, et al. Longitudinal association of total tau concentrations and physical activity with cognitive decline in a population sample. *JAMA Netw Open.* 2021;4(8):e2120398. doi:10.1001/jamanetworkopen.2021.20398

eFigure. Number of Participants by Physical Activity and Total Tau Group

eTable 1. Longitudinal Association of Continuous Variables: Physical Activity and Total Tau With Cognitive Outcomes

eTable 2. Longitudinal Association of Categorical Variables: Physical Activity and Total Tau With Global Cognitive Function

eTable 3. Longitudinal Association of Categorical Variables: Physical Activity and Total Tau With Cognitive Outcomes

eTable 4. Baseline Level of Episodic Memory, Perceptual Speed and MMSE

This supplementary material has been provided by the authors to give readers additional information about their work.

© 2021 Desai P et al. *JAMA Network Open.*

eFigure. Number of Participants by Physical Activity and Total Tau Group

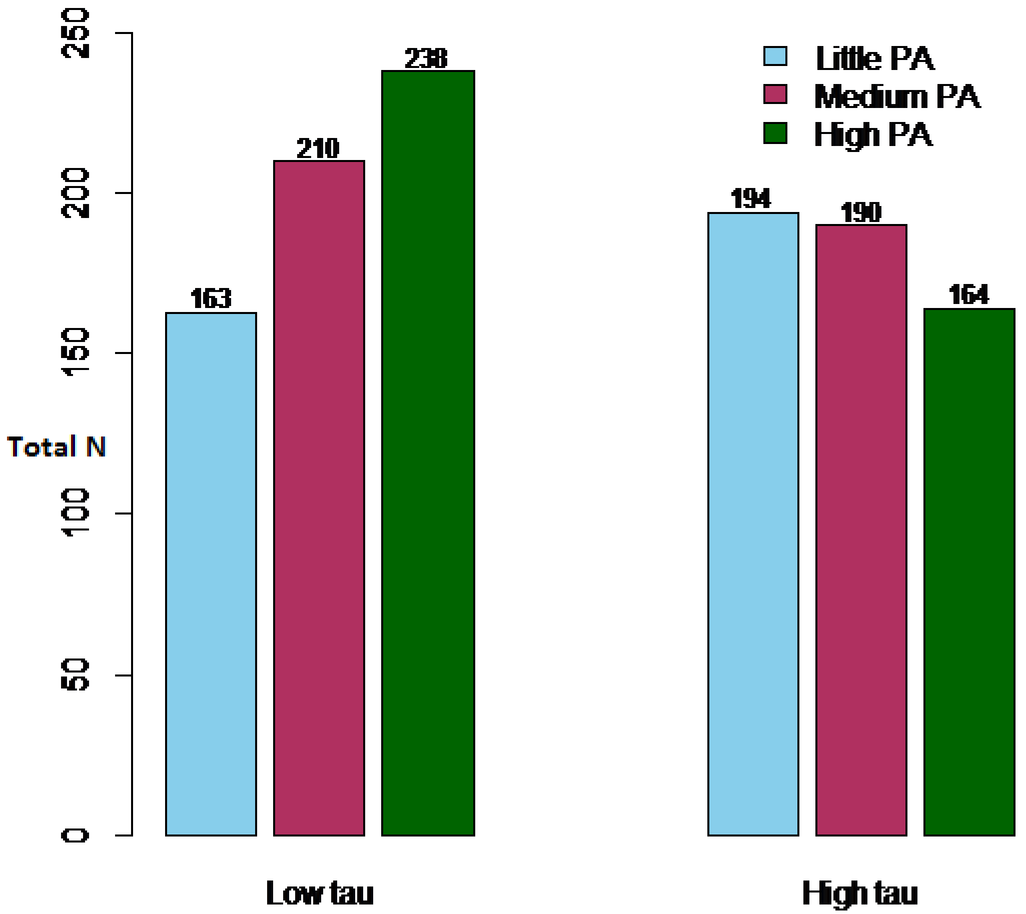

© 2021 Desai P et al. *JAMA Network Open.*

eTable 1. Longitudinal Association of Continuous Variables: Physical Activity and Total Tau With Cognitive Outcomes

	**Global Cognition (N=1159)**			**Episodic Memory (N=1150)**			**Perceptual Speed (N=1120)**			**MMSE (N=1160)**		
	β	*SE*	*p-value*	*β*	*SE*	*p-value*	*β*	*SE*	*p-value*	*β*	*SE*	*p-value*
**Intercept**	0.408	0.050	0.000**	0.273	0.064	0.000**	0.717	0.060	0.000**	0.406	0.047	0.000**
**time**	-0.057	0.009	0.000**	-0.033	0.011	0.003**	-0.069	0.009	0.000**	-0.075	0.011	0.000**
**age**	-0.021	0.004	0.000**	-0.014	0.005	0.004**	-0.031	0.004	0.000**	-0.018	0.003	0.000**
**age*time**	-0.005	0.001	0.000**	-0.005	0.001	0.000**	-0.002	0.001	0.007**	-0.005	0.001	0.000**
**Log tau**	-0.014	0.048	0.780	0.038	0.062	0.545	-0.088	0.058	0.128	-0.027	0.045	0.554
**Log tau*time**	-0.019	0.009	0.035*	-0.023	0.011	0.031*	-0.006	0.009	0.508	-0.023	0.011	0.036
**Baseline PA**	0.000	0.007	0.988	-0.002	0.009	0.872	0.012	0.009	0.161	-0.002	0.007	0.760
**PA*time**	0.001	0.001	0.502	0.001	0.002	0.540	0.002	0.001	0.198	0.000	0.002	0.943
**PA*age**	0.000	0.001	0.914	0.000	0.001	0.825	-0.001	0.001	0.484	0.000	0.001	0.891
**PA*age*time**	0.000	0.000	0.633	0.000	0.000	0.263	0.000	0.000	0.219	0.000	0.000	0.379
**Log tau*PA**	-0.002	0.009	0.786	-0.005	0.012	0.684	0.011	0.011	0.305	-0.001	0.008	0.904
**Log tau*PA*time**	0.000	0.002	0.780	0.001	0.002	0.561	0.001	0.002	0.395	-0.001	0.002	0.547

All models adjusted for age, race, sex, education, chronic medical conditions, and APOE4 and each of their interactions with time.
p<.05*, p<.01**

© 2021 Desai P et al. *JAMA Network Open.*

eTable 2. Longitudinal Association of Categorical Variables: Physical Activity and Total Tau With Global Cognitive Function

	**Low Total Tau (N=611)**			**High Total Tau (N=548)**		
	β	*SE*	*p-value*	*β*	*SE*	*p-value*
**Intercept**	0.280	0.068	0.000**	0.442	0.078	0.000**
**time**	-0.051	0.011	0.000**	-0.066	0.016	0.000**
**Medium PA**	0.131	0.059	0.027**	0.037	0.064	0.569
**High PA**	0.115	0.061	0.062	0.037	0.070	0.595
**Medium PA*time**	0.001	0.010	0.959	0.038	0.014	0.006**
**High PA *time**	0.014	0.010	0.187	0.027	0.015	0.066

*PA: Little activity (responded to at least four items and reported little activity for all responses), Medium activity (< 150 mins/ wk), High activity (≥150 mins/ wk)
Little activity is referent.
Total Tau: Low (≤ .40 pg/mL), High (> .40 pg/mL)
All models adjusted for age, race, sex, education, chronic medical conditions, and APOE4 and each of their interactions with time.
p<.05*, p<.01**

© 2021 Desai P et al. *JAMA Network Open.*

eTable 3. Longitudinal Association of Categorical Variables: Physical Activity and Total Tau With Cognitive Outcomes

	Episodic Memory						Perceptual Speed						MMSE					
	**Low Total Tau (N=607)**			**High Total Tau (N=543)**			**Low Total Tau (N=599)**			**High Total Tau (N=521)**			**Low Total Tau (N=611)**			**High Total Tau (N=549)**		
	β	*SE*	*p-value*	*β*	*SE*	*p-value*	*β*	*SE*	*p-value*	*β*	*SE*	*p-value*	*β*	*SE*	*p-value*	*β*	*SE*	*p-value*
**Intercept**	0.119	0.089	0.182	0.279	0.098	0.005**	0.668	0.085	0.000	0.819	0.093	0.000**	0.321	0.061	0.000**	0.414	0.076	0.000**
**time**	-0.021	0.014	0.129	-0.035	0.019	0.068	-0.070	0.012	0.000	-0.091	0.016	0.000**	-0.077	0.013	0.000**	-0.077	0.021	0.000**
**Medium PA**	0.139	0.078	0.075	0.088	0.081	0.281	0.090	0.074	0.223	0.003	0.076	0.964	0.067	0.053	0.207	0.013	0.062	0.831
**High PA**	0.112	0.080	0.161	-0.003	0.088	0.969	0.074	0.076	0.333	0.068	0.082	0.407	0.066	0.055	0.226	0.083	0.068	0.219
**Medium PA*time**	-0.008	0.013	0.552	0.026	0.017	0.117	0.011	0.011	0.329	0.031	0.014	0.025*	0.015	0.012	0.209	0.047	0.018	0.007**
**High PA *time**	0.007	0.013	0.613	0.036	0.018	0.049	0.016	0.011	0.134	0.031	0.015	0.034*	0.025	0.012	0.034*	0.014	0.019	0.456

*PA: Little activity (responded to at least four items and reported little activity for all responses), Medium activity (< 150 mins/ wk), High activity (>150 mins/ wk)
Little activity is referent
Total Tau: Low (≤ .40 pg/mL), High (> .40 pg/mL)
All models adjusted for age, race, sex, education, chronic medical conditions, and APOE4 and each of their interactions with time.
p<.05*, p<.01**

© 2021 Desai P et al. *JAMA Network Open.*

eTable 4. Baseline Level of Episodic Memory, Perceptual Speed and MMSE

**Episodic Memory**					
	**Estimate (95% CI)**		**Difference (95% CI)**		**% Difference**
**Low Tau**					
Little PA	0.119	(-0.056, 0.294)	Ref		Ref
Medium PA	0.258	(0.098, 0.417)	0.139	(-0.014, 0.292)	>100%
High PA	0.232	(0.085, 0.378)	0.112	(-0.045, 0.27)	94%
**High Tau**					
Little PA	0.279	(0.087, 0.471)	Ref		Ref
Medium PA	0.366	(0.181, 0.552)	0.088	(-0.072, 0.247)	32%
High PA	0.275	(0.092, 0.459)	-0.003	(-0.176, 0.169)	1%
**Perceptual Speed**					
	**Estimate (95% CI)**		**Difference (95% CI)**		**% Difference**
**Low Tau**					
Little PA	0.668	(0.501, 0.835)	Ref		Ref
Medium PA	0.758	(0.605, 0.91)	0.090	(-0.055, 0.235)	13%
High PA	0.741	(0.603, 0.88)	0.074	(-0.076, 0.223)	11%
**High Tau**					
Little PA	0.819	(0.638, 1.001)	Ref		Ref
Medium PA	0.823	(0.647, 0.998)	0.003	(-0.147, 0.153)	0.37%
High PA	0.887	(0.714, 1.06)	0.068	(-0.093, 0.229)	8%
**MMSE**					
	**Estimate (95% CI)**		**Difference (95% CI)**		**% Difference**
**Low Tau**					
Little PA	0.321	(0.202, 0.441)	Ref		Ref
Medium PA	0.388	(0.279, 0.497)	0.067	(-0.037, 0.171)	21%
High PA	0.388	(0.287, 0.488)	0.066	(-0.041, 0.174)	21%
**High Tau**					
Little PA	0.414	(0.265, 0.562)	Ref		Ref
Medium PA	0.427	(0.283, 0.571)	0.013	(-0.109, 0.136)	3%
High PA	0.497	(0.355, 0.639)	0.083	(-0.05, 0.216)	20%

*PA: Little activity (responded to at least four items and reported little activity for all responses), Medium activity (< 150 mins/ wk), High activity (≥150 mins/ wk)
Total Tau: Low (≤ .40 pg/mL), High (> .40 pg/mL)
All models adjusted for age, race, sex, education, chronic medical conditions, and APOE4.

© 2021 Desai P et al. *JAMA Network Open.*
